# UBE2C: A pan‐cancer diagnostic and prognostic biomarker revealed through bioinformatics analysis

**DOI:** 10.1002/cnr2.2032

**Published:** 2024-04-05

**Authors:** Pooya Jalali, Amir Samii, Malihe Rezaee, Arvin Shahmoradi, Fatemeh Pashizeh, Zahra Salehi

**Affiliations:** ^1^ Basic and Molecular Epidemiology of Gastrointestinal Disorders Research Centre, Research Institute for Gastroenterology and Liver Diseases, Shahid Beheshti University of Medical Sciences Tehran Iran; ^2^ Department of Hematology and Blood Transfusion School of Allied Medical Sciences, Iran University of Medical Sciences Tehran Iran; ^3^ Department of Pharmacology School of Medicine, Shahid Beheshti University of Medical Sciences Tehran Iran; ^4^ Department of Laboratory Medicine Faculty of Paramedical, Kurdistan University of Medical Sciences Sanandaj Iran; ^5^ Department of Clinical Immunology Shahid Sadoughi University of Medical Sciences Yazd Iran; ^6^ Hematology, Oncology and Stem Cell Transplantation Research Center, Tehran University of Medical Sciences Tehran Iran; ^7^ Research Institute for Oncology, Hematology and Cell Therapy, Tehran University of Medical Sciences Tehran Iran

**Keywords:** bioinformatics analysis, cancer diagnosing, pan‐cancer, TCGA, UBE2C

## Abstract

**Background:**

The diverse and complex attributes of cancer have made it a daunting challenge to overcome globally and remains to endanger human life. Detection of critical cancer‐related gene alterations in solid tumor samples better defines patient diagnosis and prognosis, and indicates what targeted therapies must be administered to improve cancer patients' outcome.

**Materials and methods:**

To identify genes that have aberrant expression across different cancer types, differential expressed genes were detected within the TCGA datasets. Subsequently, the DEGs common to all pan cancers were determined. Furthermore, various methods were employed to gain genetic alterations, co‐expression genes network and protein–protein interaction (PPI) network, pathway enrichment analysis of common genes. Finally, the gene regulatory network was constructed.

**Results:**

Intersectional analysis identified UBE2C as a common DEG between all 28 types of studied cancers. Upregulated UBE2C expression was significantly correlated with OS and DFS of 10 and 9 types of cancer patients. Also, UBE2C can be a diagnostic factor in CESC, CHOL, GBM, and UCS with AUC = 100% and diagnose 19 cancer types with AUC ≥90%. A ceRNA network constructed including UBE2C, 41 TFs, 10 shared miRNAs, and 21 circRNAs and 128 lncRNAs.

**Conclusion:**

In summary, UBE2C can be a theranostic gene, which may serve as a reliable biomarker in diagnosing cancers, improving treatment responses and increasing the overall survival of cancer patients and can be a promising gene to be target by cancer drugs in the future.

## 1 INTRODUCTION

The diverse and complex attributes of cancer have made it a daunting challenge to overcome globally and remains to endanger human life. As a leading cause of mortality, each year new cases are added, intensifying the burden of the disease. The annual prevalence of cancer was approximately 20 million newly affected cases in 2020, resulting in the deaths of almost 10 million people, and it is anticipated to increase by nearly 50% over the next two decades.^1^ Despite advances in treatment, accelerated incidence rates indicate that more cancer preventive measures should be implemented to counteract the detrimental impacts of cancer on patients' survival. Therefore, the identification of specific and sensitive biomarkers for the diagnosis and treatment of cancer can improve timely diagnosis, develop tailored therapy and improve overall patient outcomes.

Since the underlying molecular mechanisms and genetic changes involved in the development and progression of various types of cancer are very heterogeneous, investigating cancer pathogenesis is very challenging. Numerous etiologic factors affecting cancer pathogenesis including genetic alterations and epigenetic modifications that involve gene mutation and methylation lead to cancer initiation and progression.^2^ Therefore, acquiring a thorough comprehension of cancer pathogenesis at the molecular level is essential in order to improve designing novel preventive or therapeutic approaches for better cancer management.

As a ubiquitous tool in molecular biology, RNA sequencing has been introduced as a novel method, which enables researchers in many aspects of cancer research studies and provides insight into the detection of genomic aberrations and transcript isoforms such as mutation and copy number alteration. It also facilitates the identification of alternative gene‐spliced transcripts, posttranscriptional modifications, and gene expression alternations.^3^, ^4^ In recent years, next‐generation sequencing (NGS) technology with high accuracy and sensitivity has played an essential role in the understanding of the altered genetic pathways involved in human cancer and genome sequencing has made simultaneous screening of several mutated genes in multiple cancers feasible.^5^ Detection of critical cancer‐related gene alterations in solid tumor samples better defines patient diagnosis and prognosis, and indicates what targeted therapies must be administered to improve cancer patients' outcome.^6^ Many studies have enumerated NGS applications for analysis of genetic variation and tumor mutation burden in various solid tumors such as colorectal cancer, gastric cancer, and breast cancer.^7^, ^8^, ^9^ Although all cancers are molecularly distinct, many share common driver mutations.

Pan‐cancer analysis employs NGS for examination of frequently mutated genes and other genomic abnormalities that are common among many cancer types, regardless of the tumor origin.^10^ Interestingly, pan‐cancer analysis has revealed that some cancers arising from different organs exhibit molecular similarities, while certain cancers originating from the same tissue can have distinct genomic profiles.^11^, ^12^ Numerous studies have been conducted to explore the expression level of one specific gene in various cancers.^13^, ^14^, ^15^ Recently, the prognostic significance, and immunological functions of ubiquitin‐conjugating enzyme 2C (UBE2C) has been studied across various tumor types.^16^, ^17^

In the current study, we aimed to detect differentially expressed gene among various cancer types. We also examined the candidate gene prognostic/diagnostic value, its correlation with immune signatures, and the associated competing endogenous RNA (ceRNA) network. Our study highlights the clinical significance and potential regulatory mechanisms of UBE2C as an upregulated biomarker in approximately all types of tumors. These findings suggest that targeting UBE2C could enhance the effectiveness of cancer therapies beyond a single cancer type, particularly in tumors with a similar genetic profile.

## 2 MATERIALS AND METHODS

**FIGURE 1:**
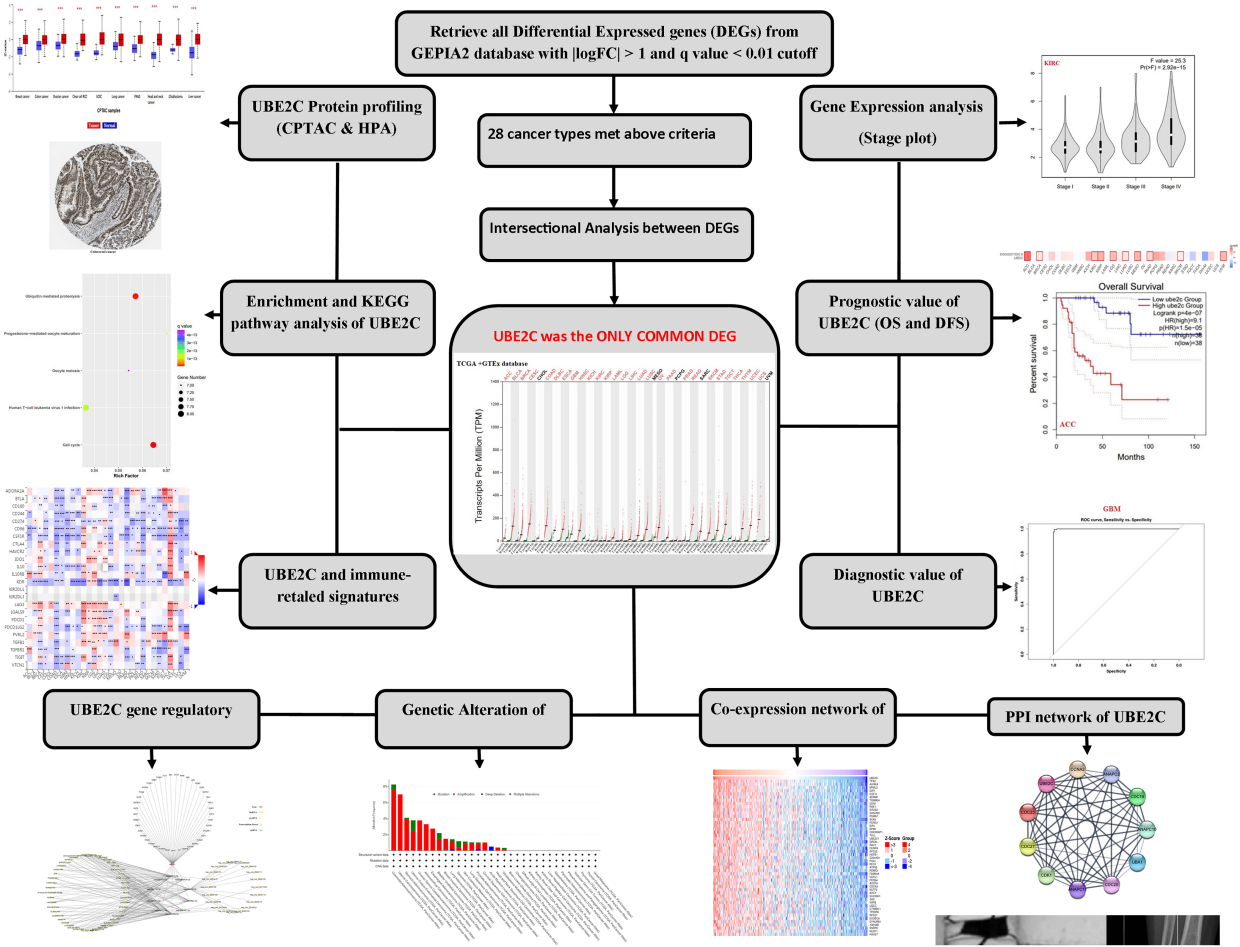
Study workflow for pan‐cancer analysis of UBE2C. After intersectional analyzing of TCGA cancer types, UBE2C was found as a gene, which is significantly differentiated in all 28 types of cancer. Then, via various bioinformatics databases, we comprehensively and systematically studied the roles of UBE2C in all types of cancer.

Initially, we identified DEGs in pan‐cancers using TCGA datasets. Next, we determined DEGs shared across all cancers. We employed diverse techniques to uncover genetic alterations, co‐expression gene networks, protein–protein interaction (PPI) networks, correlations with immune related gene signatures, enriched pathways, and protein profiles. Lastly, we constructed a competing endogenous RNA (ceRNA) network involving long noncoding RNA (lncRNA), micro RNA (miRNA), circular RNA (circRNA), and mRNA interactions (Figure 1).

### 2.1 Identification of differentially expressed genes

The GEPIA2 platform (http://gepia2.cancer-pku.cn/)^18^ was utilized to identify differentially expressed genes (DEGs) that are associated with pan cancers among the high throughput RNA‐Seq data. The data was obtained from TCGA and GTEx database, which have tumor tissues, adjacent normal tissues, and normal tissues in normal individuals. In this study, DEGs that exhibited upregulation or downregulation pattern, with an absolute value of |log2 Fold Change (FC)| >1 and an adjusted *p*‐value <.01, were considered statistically significant.

### 2.2 Investigating diagnostic and prognostic value of the candidate common gene

To assess the diagnostic properties of the identified common differentially expressed gene, the gene expression data was extracted from the OncoDB database (https://oncodb.org/), which provides TCGA data.^19^ Subsequently, sensitivity, specificity, and the Area under the Receiver Operating Characteristic Curve (AUC) were computed to determine the diagnostic efficacy of the common DEG for distinguishing between tumor tissues and their normal counterparts. This evaluation was performed using the biomarker analysis tools available at https://analysistools.cancer.gov/biomarkerTools. Additionally, the prognostic values of the candidate gene were examined thorough survival analyses using GEPIA2 database pan cancer TCGA data; both tumor and adjacent normal tissues were utilized to perform the analysis.

### 2.3 Co‐expression analysis and PPI network

In the present study, the LinkedOmics tool^20^ was used to obtain positively and negatively correlated genes with the identified common DEGs in the candidate cancers. In addition, we employed the STRING database (https://string-db.org) to construct a PPI network for the common DEGs, which was subsequently visualized using the Cytoscape software (version 3.9.1; https://cytoscape.org). Additionally, the GEPIA2 database was utilized to identify the top genes that exhibit a similar expression pattern to the common DEGs.^18^ Subsequently, an intersection analysis was conducted to determine the proteins that interact with common DEGs and the top 100 genes with a similar expression pattern.

### 2.4 Gene set enrichment analysis

To assess the functional annotation and pathway enrichment of the common DEGs, we conducted a gene enrichment analysis using the Enrichr database (https://maayanlab.cloud/Enrichr/).^21^ This involved evaluating the gene oncology (GO) categories, which encompass biological processes (BP) and molecular functions (MF), as well as examining the Kyoto Encyclopedia of Genes and Genomes (KEGG). The enrichment analyses were conducted by considering the common DEGs and their associated genes.

### 2.5 Genetic alteration

The cBioPortal (https://www.cbioportal.org/) was used to obtain the genetic alteration data of common DEGs. The cBioPortal is a comprehensive online platform designed to allow exploration, visualization, and analysis of multidimensional cancer genomics data.^22^ In this study, we utilized the “Cancer Types Summary” module within cBioPortal to access information on the alteration frequency, mutation type, and copy number alteration of the common DEGs across all 33 tumors in the TCGA dataset. Specifically, by selecting the “TCGA Pan Cancer Atlas Studies” within the “Quick select” section, we were able to query and identify the genetic alteration characteristics specific to the common DEGs.

### 2.6 Protein expression profiling

To demonstrate the manner, in which mRNA of common DEGs and protein expression data can vary across diverse classifications of human cancers the human protein atlas (HPA) database (https://www.proteinatlas.org) was employed. Moreover, supplementary immunohistochemistry images of common DEGs encoded proteins derived from cancerous tissues were also acquired for further investigation. Further, we obtained pan‐cancer protein expression of selected DEGs in tumor tissue from different cancers and in adjacent normal tissues, the Clinical Proteomic Tumor Analysis Consortium (CPTAC) module of UALCAN was utilized (http://ualcan.path.uab.edu/analysis.html).

### 2.7 Immune interactions

This study focuses on examining the correlations between the expression of common DEGs and immune‐related indicators pertaining to immune cells, immunoinhibitory and immunostimulatory factors, and HLA molecules in the context of human cancers using TISIDB. [40].

### 2.8 Transcription factor, miRNA, lncRNA, ceRNA, and ceRNA network

Bioinformatics online tool hTFtarget (http://bioinfo.life.hust.edu.cn) has compiled detailed transcription factor (TF)—target regulations from massive ChIP‐Seq data of human TFs.^23^ In this study, hTFtarget was utilized to identify TFs of Common DEGs. Tfcancer is an online tool, which includes 3136 experimentally supported associations between 364 TFs and 33 TCGA cancers by manually curating more than 1800 studies.^24^ We used Tfcancer database to validate the results from hTFtarget. Detecting of the miRNAs targeting common DEGs was conducted using miRTarBase, an integrated web database for determining miRNA‐target interactions.^25^ Furthermore, to identify lncRNAs associated with the DEGs miRNAs, the miRNet database, a miRNA‐centric network visual analytics platform (https://www.mirnet.ca/) was utilized. To identify circRNAs that regulate common DEGs, circBank database^26^ was utilized. CircRNAs with Tot score >1000 were selected for further analysis. Using Cytoscape,^27^ a ceRNA network was established, encompassing common DEGs, their TFs, miRNAs targeting DEGs, and lncRNAs and circRNAs, which act as sponges for the identified miRNAs. Also, hub nodes of UBE2C ceRNA network were identified utilizing plug‐in Centiscape 2.2.

## 3 RESULTS

### 3.1 Pan‐cancer gene expression analysis determined UBE2C as a common differentially expressed gene positively correlated with cancer stage

**FIGURE 2:**
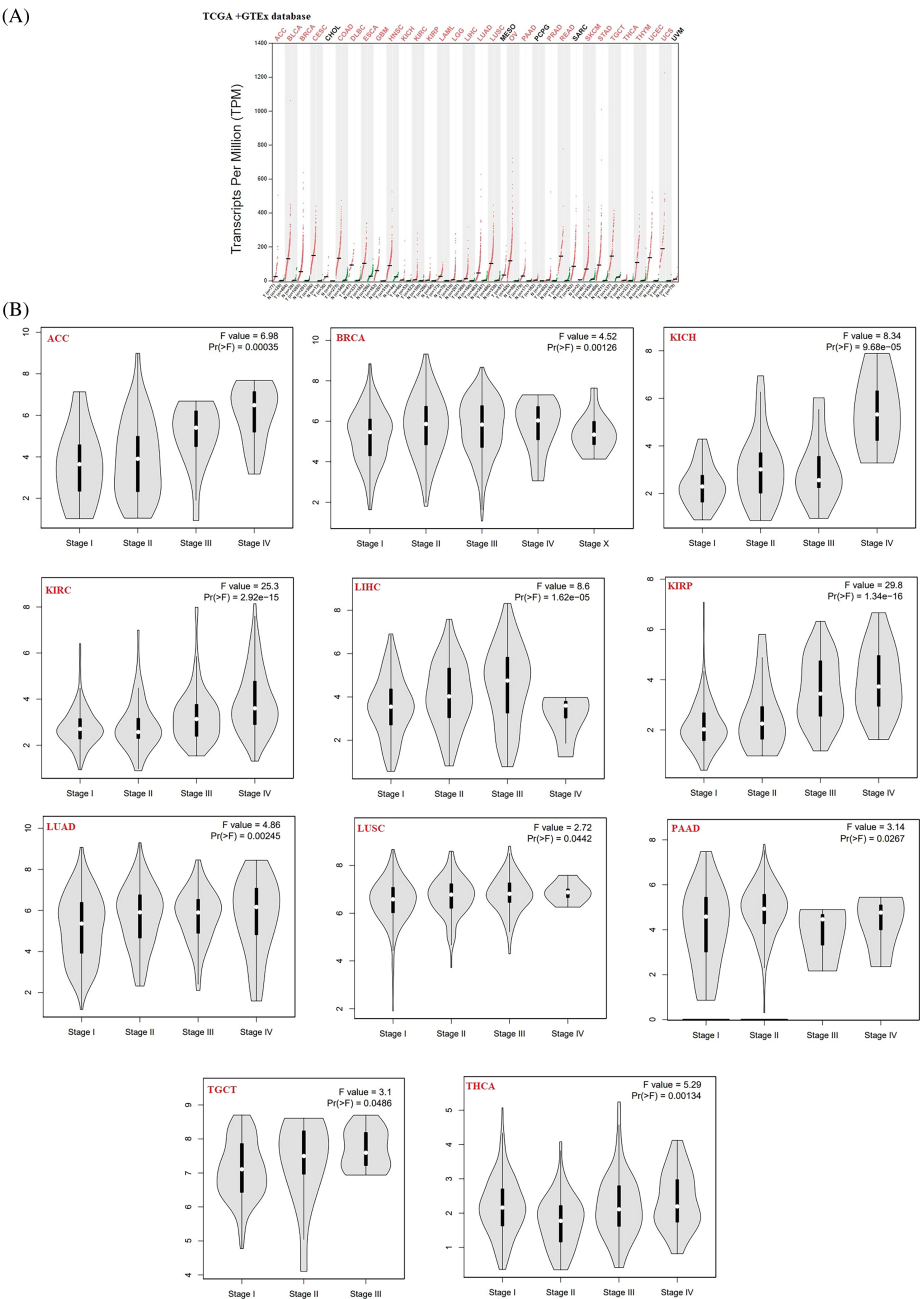
UBE2C gene expression in different tumors and pathological stages. (A) UBE2C gene expression pan cancer analyzing showed that UBE2C was upregulated in 28 types of cancer. (B) Using TCGA data, UBE2C expression level was significantly correlated with pathological stages of cancers including ACC, BRCA, KICH, KIRC, KIRP, LIHC, LUAD, LUSC, PAAD, TGCT, and THCA.

Out of the 33 cancers in the TCGA datasets, by *q*‐value cutoff = 0.01 and |LogFC| cutoff >1, cholangiocarcinoma (CHOL), Mesothelioma (MESO), pheochromocytoma and paraganglioma (PCPG), sarcoma (SARC), and uveal melanoma (UVM) did not meet these criteria and were therefore excluded from the subsequent analysis. The remaining 28 cancers‐associated DEGs were selected for further investigation. Intersectional analysis identified UBE2C as a common DEG between all 28 types of studied cancers. Pan‐cancer analysis was performed to compare expression levels of the UBE2C gene between tumor and normal tissues. By *q‐*value cutoff = 0.01 and |LogFC| cutoff = 1, UBE2C gene was upregulated in 28 types of cancer (Figure 2A). Also, “Expression Analysis‐Box Plots” module showed that by *p*‐value cutoff = 0.01 and |LogFC| cutoff = 1, among 31 types of cancers, UBE2C was upregulated in 30 cancers (Supplementary Figure 1). The differential expression of UBE2C in different tumor types suggested that UBE2C has various regulatory mechanisms in different tumor types. Furthermore, UBE2C pan‐cancer Stage plot analyzing was performed. There was a significant correlation between UBE2C expression level and the pathological stages of cancers including adrenocortical carcinoma (ACC, *p*‐value = .00035), Breast Cancer (BRCA, *p*‐value = .00126), Kidney Chromophobe (KICH, *p*‐value = 9.68e‐05), Kidney renal clear cell carcinoma (KIRC, *p*‐value = 2.92e‐15), Kidney renal papillary cell carcinoma (KIRP, *p*‐value = 1.36e‐16), Liver hepatocellular carcinoma (LIHC, *p*‐value = 1.62e‐5), Lung adenocarcinoma (LUAD, *p*‐value = .00245), Lung squamous cell carcinoma (LUSC, *p*‐value = .442), Pancreatic adenocarcinoma (PAAD, *p*‐value = .0267), Tenosynovial giant cell tumor (TGCT, *p*‐value = .0486), and Thyroid carcinoma (THCA, *p*‐value = .00134) (Figure 2B).

### 3.2 UBE2C expression is correlated with poor prognosis

**FIGURE 3:**
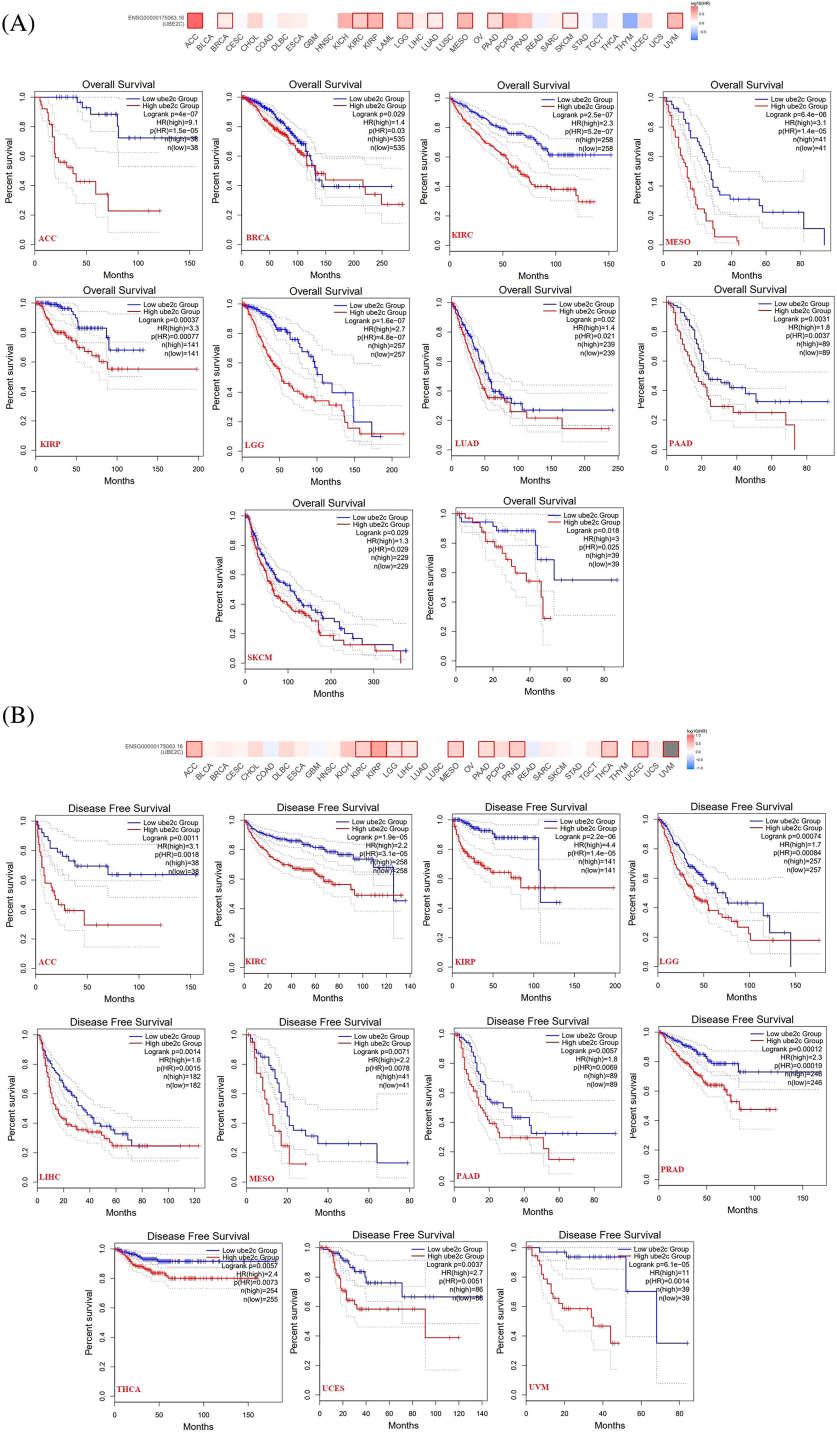
The correlation between UBE2C gene expression and prognosis of patients with different tumors. There was a significant correlation between upregulated UBE2C expression and Overall survival (A) and Disease‐free survival (B) of patients with tumors.

Pan‐cancer prognostic value of UBE2C was investigated using dataset from GEPIA2. Upregulated UBE2C expression was significantly correlated with poor prognosis and Overall survival (OS) of patients with ACC, BRCA, KIRC, KIRP, Low grade glioma (LGG), LUAD, MESO, PAAD, and Skin cutaneous melanoma (SKCM) (Figure 3A). Also, upregulated UBE2C expression was significantly correlated with poor prognosis and disease free survival (DFS) of patients with ACC, KIRC, KIRP, LGG, LIHC, MESO, PAAD, Prostate adenocarcinoma (PRAD), THCA, Uterine corpus endometrial carcinoma (UCEC), and UVM (Figure 3B).

### 3.3 UBE2C plays as a diagnostic factor

To study the diagnostic value of UBE2C in different cancers, expression levels of UBE2C in cancer and normal patients in different cancers were retrieved using OncoDB database. Receiver operating characteristic (ROC) curve of UBE2C in various cancers showed that UBE2C can be a diagnostic factor in cervical squamous cell carcinoma (CESC, AUC = 100%), CHOL (AUC = 100%), glioblastoma (GBM, AUC = 100%), uterine carcinosarcoma (UCS, AUC = 100%), ACC (AUC = 93.9%), bladder urothelial carcinoma, (BLCA, AUC = 94.6%), BRCA (AUC = 97%), COAD (AUC = 97.9%), Esophageal carcinoma (ESCA, AUC = 95.7%), KICH (AUC = 95.9%), KIRC (AUC = 94.2%), KIRP (AUC = 94%), LGG (AUC = 95.9%), LIHC (AUC = 97.5%), LUAD (AUC = 97.7%), LUSC (AUC = 99.8%), ovarian serous cystadenocarcinoma (OV, AUC = 99.8%), PAAD (AUC = 99.2%), rectum adenocarcinoma (READ, AUC = 98.9%), SKCM (AUC = 90%), stomach adenocarcinoma (STAD, AUC = 94.5%), TGCT (AUC = 99.2%), and UCEC (AUC = 97.3%) (Supplementary Figure 2).

### 3.4 CDCA3 defined as a UBE2C consistently co‐expressed gene across 30 cancer types

**FIGURE 4:**
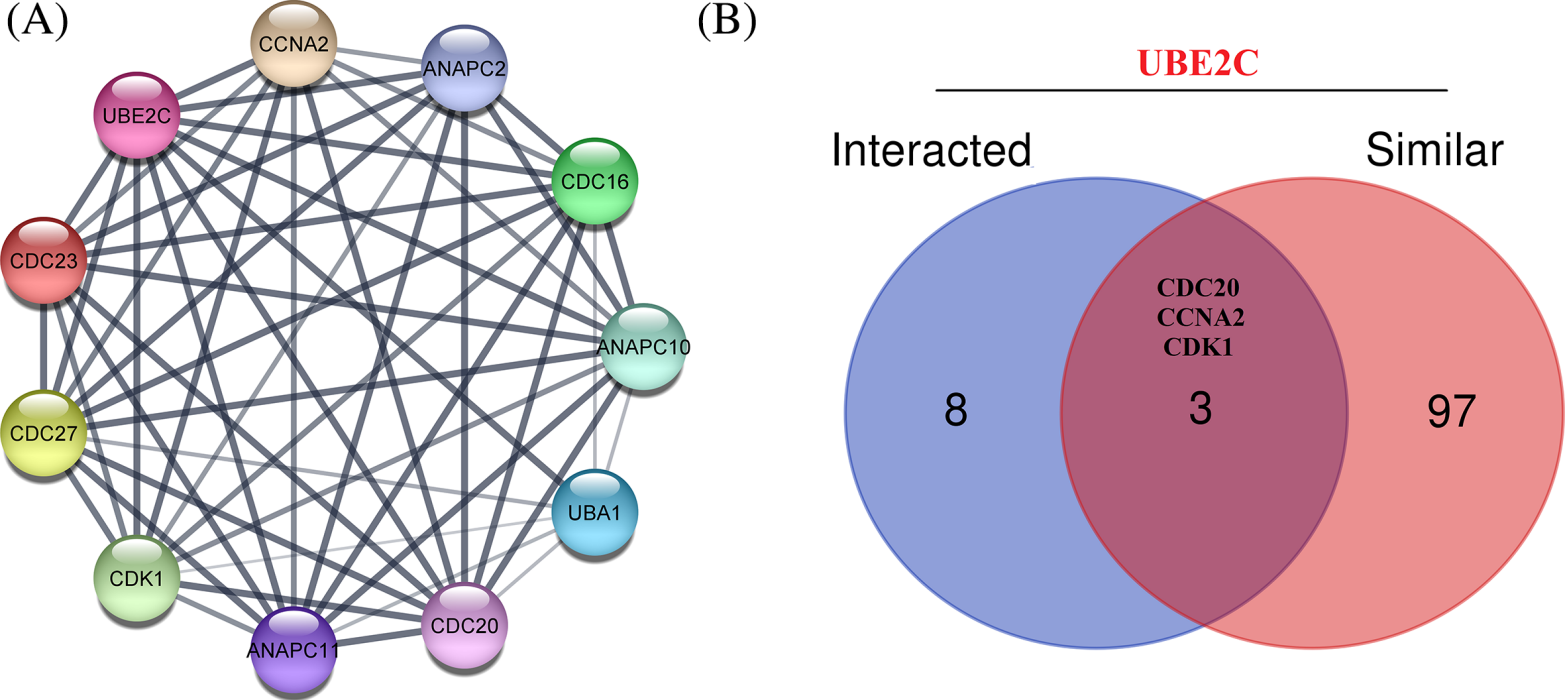
Protein–protein interaction network and similar and interacted genes. (A) PPI network of UBE2C. (B) Intersection analysis of UBE2C similar and interacted genes.

UBE2C co‐expressed genes in different types of cancer were retrieved using LinkedOmics [30]. Furthermore, by intersectional analysis common genes, which strongly correlated with selected DEGs (rho >0.6) in all types of cancer were identified. CDCA3 was found as a UBE2C correlated gene, which is common in 30 types of TCGA cancers (Supplementary File 1). By using STRING database, a network of 10‐UBE2C binding protein interactions was constructed (Figure 4A). The network had 11 nodes and 52 edges. Furthermore, top 100 genes that had similar expression pattern with UBE2C expression were selected using GEPIA2. Three genes including CDC20, CCNA2, and CDK1 were found as common genes between UBE2C similar and interacted genes (Figure 4B).

GO and KEGG pathway enrichment analysis showed that UBE2C and its interacting genes significantly enriched in 21 Biological processes (BPs) and 4 Molecular functions (MFs) and 4 KEGG pathways (*q*‐value <0.01) (Table 1). The most significant biological process and molecular function were receptor Anaphase‐Promoting Complex‐Dependent Catabolic Process (GO:0031145; *q*‐value = 1.33E‐24, RF = 0.44), and Ubiquitin‐Like Protein Transferase Activity (GO:0019787; *q*‐value = 0.005, RF = 0.0125). Further, the most significant KEGG pathway was Cell cycle (*q*‐value = 5.07E‐16, RF = 0.065).

**TABLE 1:** Gene set enrichment analysis of UBE2C and its interacting genes.

Biological Process	Gene number	Rich factor	*q*‐value
Anaphase‐promoting complex‐dependent catabolic process (GO:0031145)	9	0.44	1.33E‐24
Regulation of meiotic cell cycle (GO:0051445)	8	0.4	1.90E‐21
Protein K11‐linked ubiquitination (GO:0070979)	7	0.24	7.81E‐17
Regulation of mitotic cell cycle (GO:0007346)	8	0.06	8.73E‐15
Proteasome‐mediated ubiquitin‐dependent protein catabolic process (GO:0043161)	9	0.03	7.41E‐14
Regulation of cell cycle (GO:0051726)	8	0.03	6.79E‐12
Protein polyubiquitination (GO:0000209)	7	0.03	1.13E‐10
Regulation of mitotic metaphase/anaphase transition (GO:0030071)	5	0.1	4.74E‐10
Positive regulation of mitotic sister chromatid separation (GO:1901970)	4	0.27	8.68E‐10
Positive regulation of metaphase/anaphase transition of cell cycle (GO:1902101)	3	0.27	2.15E‐07
Positive regulation of mitotic metaphase/anaphase transition (GO:0045842)	3	0.27	2.15E‐07
Positive regulation of mitotic cell cycle phase transition (GO:1901992)	4	0.06	2.80E‐07
Mitotic cell cycle phase transition (GO:0044772)	4	0.04	2.57E‐06
Positive regulation of mitotic nuclear division (GO:0045840)	3	0.09	6.65E‐06
Positive regulation of synaptic plasticity (GO:0031915)	2	0.33	3.19E‐05
Positive regulation of synapse maturation (GO:0090129)	2	0.25	5.24E‐05
Positive regulation of ubiquitin protein ligase activity (GO:1904668)	2	0.25	5.24E‐05
Regulation of synapse maturation (GO:0090128)	2	0.2	7.96E‐05
Regulation of ubiquitin protein ligase activity (GO:1904666)	2	0.12	2.27E‐04
Positive regulation of ubiquitin‐protein transferase activity (GO:0051443)	2	0.07	5.56E‐04
Positive regulation of cellular component organization (GO:0051130)	2	0.02	0.009298
Molecular function			
Ubiquitin‐like protein transferase activity (GO:0019787)	3	0.0125	0.005244
Ubiquitin‐protein transferase activity (GO:0004842)	3	0.007282	0.008713
Phosphatase binding (GO:0019902)	2	0.017857	0.008713
Protein phosphatase binding (GO:0019903)	2	0.017391	0.008713
KEGG pathway			
Cell cycle	8	0.064516	5.07E‐15
Ubiquitin mediated proteolysis	8	0.057143	6.87E‐15
Progesterone‐mediated oocyte maturation	7	0.07	1.23E‐13
Human T‐cell leukemia virus 1 infection	8	0.03653	1.31E‐13
Oocyte meiosis	7	0.054264	4.58E‐13

### 3.5 High burden of missense mutation in UBE2C is significantly correlated with poor prognosis and disease‐specific survival

**FIGURE 5:**
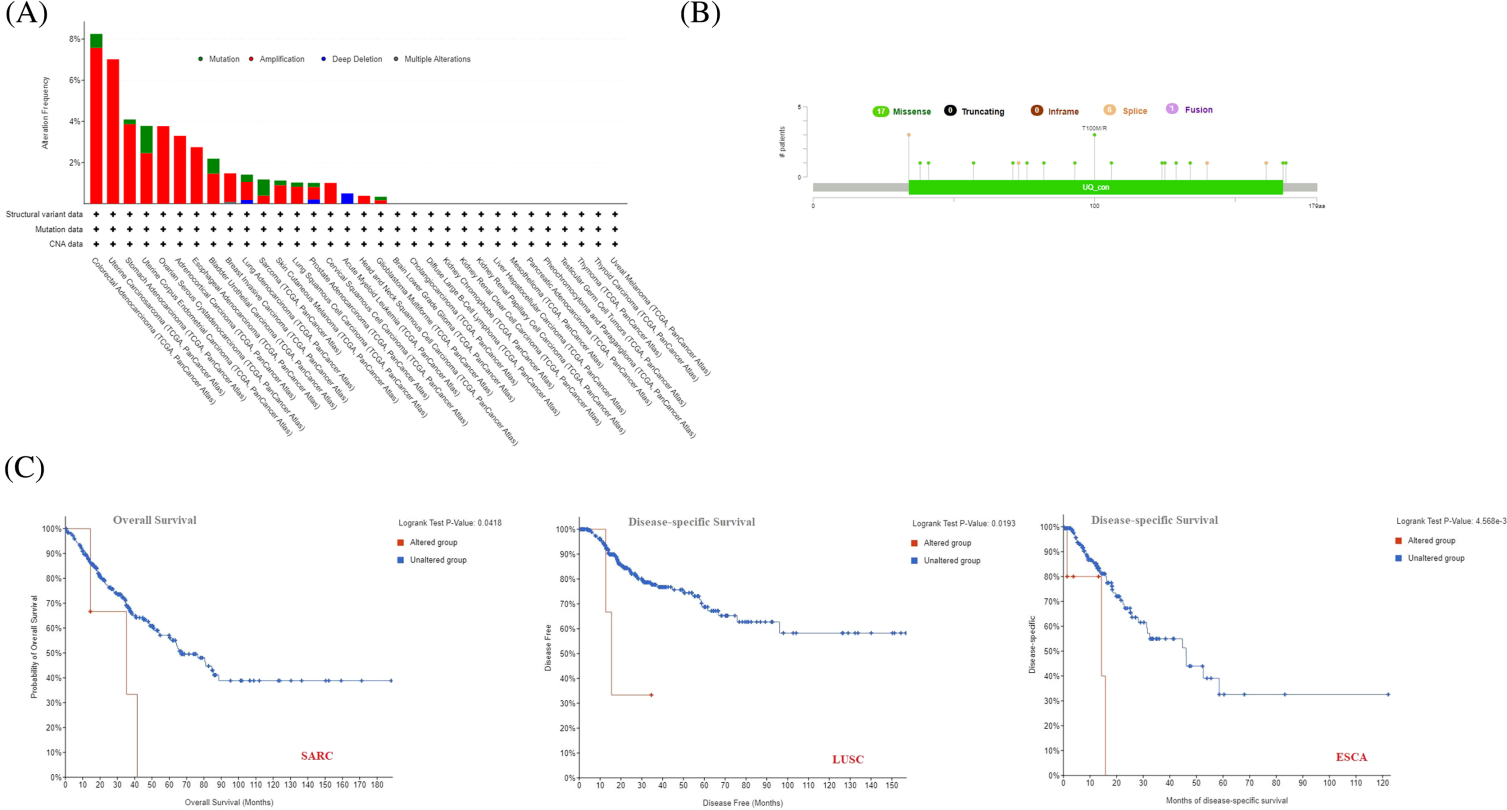
Genetic alteration features of UBE2C in various cancers. (A) The alteration frequencies and mutation types of UBE2C in various cancers in TCGA. UBE2C has the highest mutation frequency in patients with colorectal adenocarcinoma. (B) The alteration frequencies and mutation sites of UBE2C. The main type of genetic alteration in UBE2C was Missense mutation. (C) The potential correlation between UBE2C alteration and overall survival and disease‐specific survival. UBE2C alteration was significantly correlated between disease‐specific survival of patients with ESCA and LUSC and overall survival of patient with SARC.

To investigate the correlation between UBE2C genetic alterations among various cancers, the Cbioportal tool was used to identify genetic alterations in data extracted from TCGA dataset. UBE2C has the highest mutation frequency in patients with colorectal adenocarcinoma (~8%). The genetic alteration frequency of UBE2C in uterine carcinosarcoma is nearly as high. Notably, all patients with UCS, OV, ESCA, CESC, and head and neck squamous cell carcinoma (HNSC) had amplification of the UBE2C gene, which showed an alteration frequency of ~7%, ~4%, ~3%, ~2%, and ~1%, respectively (Figure 5A). Also, deep deletion was seen among all LAML patients with genetic alterations in UBE2C, which showed an alteration frequency of nearly ~1%. UBE2C mutation types, sites and case numbers are shown in Figure 5B. The main type of genetic alteration in UBE2C was missense mutation. In patients with ESCA and LUSC, UBE2C alteration was significantly correlated with poor prognosis and Disease‐specific Survival. Also, UBE2C alteration was significantly correlated with poor prognosis and Overall Survival of patients with SARC (*p*‐value <.05) (Figure 5C).

### 3.6 Protein expression profiling

**FIGURE 6:**
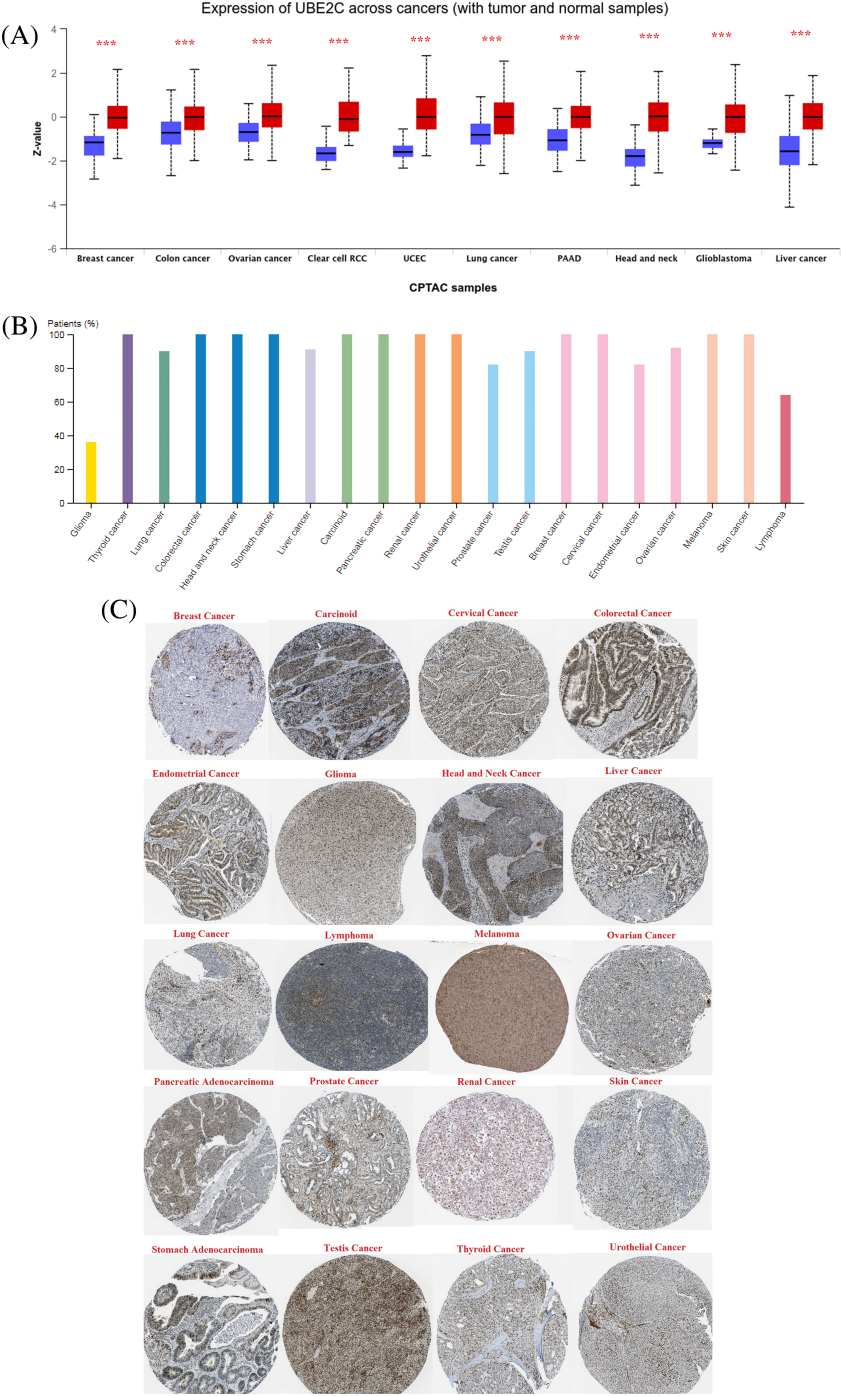
Protein expression of UBE2C in different types of cancer. (A) UBE2C protein level was upregulated in breast cancer, colon cancer, ovarian cancer, clear cell renal cell carcinoma, UCEC, lung cancer, PAAD, head and neck cancer, glioblastoma, and liver cancer utilizing CPTAC dataset. (B) Although the immunohistochemical staining results from HPA database for different cancer types showed strong strain, several cases of gliomas were weakly stained or negative. (C) Pathological section images of cancer types showed upregulation of UBE2C in 20 types of cancer.

To study UBE2C protein expression levels in different types of cancer, we used the CPTAC and HPA databases. High UBE2C protein expression levels were seen in all cancer types (Figure 6A). Although the immunohistochemical (IHC) staining results from HPA database for different cancer types showed strong strain, several cases of gliomas were weakly stained or negative (Figure 6B). Aberrant protein expression of UBE2C was detected in 20 types of tumor tissues (Figure 6C).

### 3.7 UBE2C expression correlates with immune signatures across cancers

**FIGURE 7:**
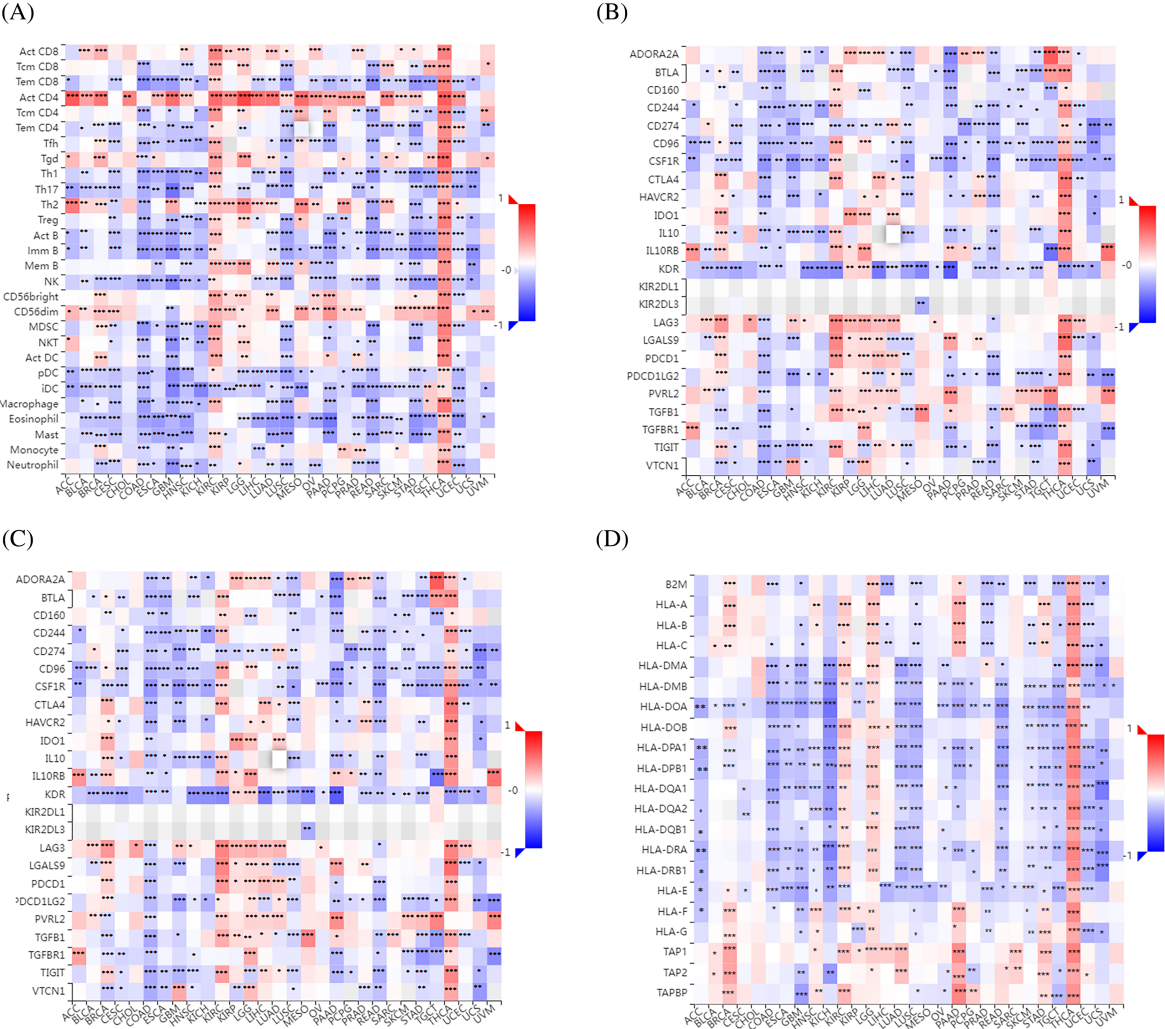
Correlation between UBE2C expression and immune‐related signatures. There were strong correlations between UBE2C expression and immune cells (A), immunoinhibitors (B), immunstimulators (C), and HLA molecules (D) across human cancers.

We studied associations between UBE2C gene expression and immune‐related signatures including immune cells, immunoinhibitors, immunstimulators and HLA molecules across human cancers through TISIDB (http://cis.hku.hk/TISIDB/) [40]. There was a strong and significant correlation between UBE2C gene expression and all immune cells in THCA. Most immune cells had significant correlation with UBE2C gene expression in COAD, GBM, KIRC, LUSC, PAAD, READ, STAD, and UCEC. Also, in most of the cancers, there was a significant correlation between UBE2C gene expression and CD4+ T cell (Act CD4), effector memory CD8+ T cell (Tem‐CD8), type 1 T helper cell (Th1), type 2 T helper cell (Th2), immature B cell (Imm B), natural killer cell (NK), CD 56dim natural killer cell (CD56dim), plasmacytoid dendritic cell (pDC), immature dendritic cell (iDC), eosinophil, and mast cell (Figure 7A). Further, almost all immunoinhibitors had significant correlation with UBE2C expression in COAD and THCA. Also, in most of the cancers, there was a significant correlation between UBE2C gene expression and CD96, CD274, CSF1R, KDR, and PDCD1LG2 (Figure 7B). There was a significant correlation between UBE2C expression and most of the immunostimulators in COAD, LUSC and THCA. Also, in most of the cancers, there was a significant correlation between UBE2C gene expression and C10orf54, CXCL12, IL6R, MICB, PVR, THEM173, and TNFSF13 (Figure 7C). Additionally, the correlation analysis was performed between UBE2C expressions and MHC molecules. All MHC molecules had significant correlation with THCA. In most of the cancers, there was a significant correlation between UBE2C gene expression and HLA‐DMB, HLA‐DOA, HLA‐DPA1, HLA‐PDB1, HLA‐DRA, and HLA‐E (Figure 7D).

### 3.8 CeRNA network construction

In this study, 314 TFs of UBE2C were identified using hTFtarget. Among them, 41 TFs were validated in TCGA cancers based on literature (Supplementary File 2).

Due to large number of miRNAs associated with UBE2C, which makes it challenging to comprehensively study their interactions with lncRNAs, we focused on miRNAs validated by experimental studies and available in the miRTarBase database. Ten miRNAs, including *hsa‐miR‐196a‐5p*, *hsa‐miR‐615‐3p*, *hsa‐miR‐671‐5p*, *hsa‐miR‐17‐5p*, *hsa‐miR‐20a‐5p*, *hsa‐miR‐381‐3p*, *hsa‐miR‐24‐3p*, *hsa‐miR‐193b‐3p*, *hsa‐miR‐16‐5p*, and *hsa‐miR‐140‐3p* were found as miRNAs associated with UBE2C.

To identifying potential circular RNAs regulating selected miRNAs, circbank database was used. With tot score >1000 cutoff, 21 circRNAs were selected, including *hsa_circ_0052415*, *hsa_circ_0079934*, *hsa_circ_0079934*, *hsa_circ_0075924*, *hsa_circ_0017040*, *hsa_circ_0059100*, *hsa_circ_0059101*, *hsa_circ_0049106*, *hsa_circ_0049105*, *hsa_circ_0049109*, *hsa_circ_0049110*, *hsa_circ_0049111*, *hsa_circ_0049112*, *hsa_circ_0049113*, *hsa_circ_0049104*, *hsa_circ_0049115*, *hsa_circ_0049116*, *hsa_circ_0049120*, *hsa_circ_0049099*, *hsa_circ_0049103*, and *hsa_circ_0049108*.

Furthermore, to studying lnRNAs regulating selected miRNAs, miRNet database was used. A total of 316 lncRNAs were identified (Supplementary File 3). By intersectional analysis of lncRNAs, NEAT1 was found as a common lncRNA regulating all identified miRNAs. Also, 48 lncRNAs were found having interaction with ≥3 selected miRNAs.

**FIGURE 8:**
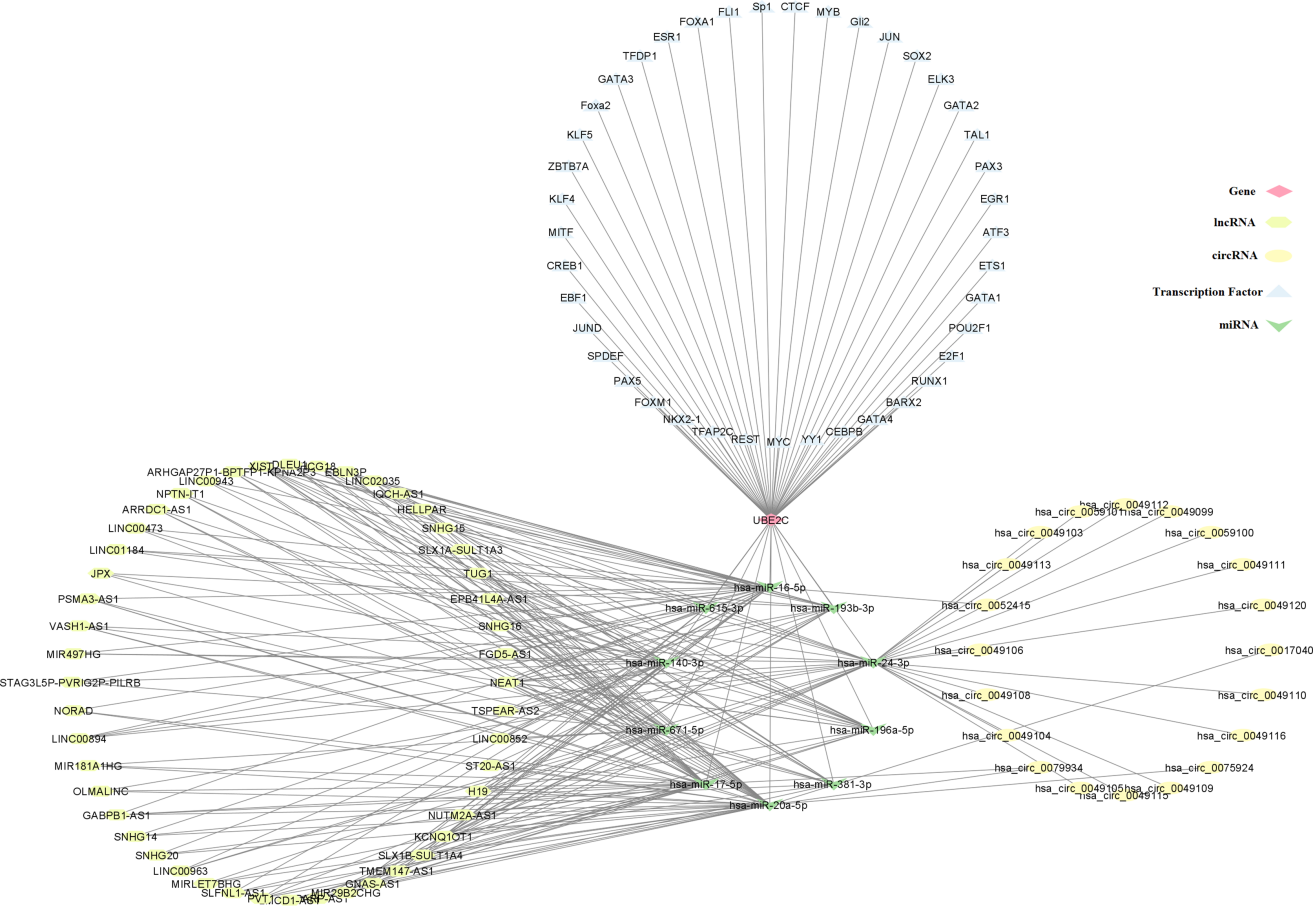
The ceRNA network of UBE2C. The ceRNA network includes UBE2C, its 41 TFs, 10 shared miRNAs, and 21 circRNAs and 48 lncRNAs.

Finally, ceRNA network constructed, including UBE2C, 41 TFs, and 10 shared miRNAs, and 21 circRNAs and 48 lncRNAs (Figure 8).

## 4 DISCUSSION

In the current study, pan cancer analysis identified UBE2C as a common DEG nearly among all types of cancer, which was correlated with poor prognosis and have a crucial diagnostic value in many cancer types.

A comprehensive pan‐cancer analysis of UBE2C expression in a wide array of tumors revealed that UBE2C is upregulated in 28 different cancers, which is in alignment with earlier findings as stated in previous reports.^16^, ^17^ Consistent with our finding, previous studies reported that the UBE2C mRNA expression was higher in various cancers including hepatocellular,^28^ esophageal squamous cell,^29^ adrenocortical carcinoma,^30^ gastric,^31^ breast,^32^ and lung cancers.^33^ UBE2C is a part of the ubiquitin proteasome system, having a key function in the regulation of cell cycle progression via the degradation of mitotic cyclins to control the M phase to the G1 phase transition and also engaging in mitotic spindle checkpoint control.^34^, ^35^ Upregulated UBE2C expression disturbs the ubiquitination system required for protein degradation, leading to aberrant cell proliferation as a common feature of malignant transformation in a wide range of malignancies.^36^, ^37^, ^38^ Taken together, higher UBE2C gene expression appeared to be a useful prognostic indicator associated with poor prognosis and higher frequencies of tumor metastasis and progression.^28^, ^29^, ^30^, ^31^, ^32^, ^33^

Furthermore, we indicated that the expression level of UBE2C has altered in different pathological stages that positively correlated with cancer stage and the results showed higher expression of UBE2C in the late stages of cancer compared to early stages including ACC, BRCA, KICH, KIRC, KIRP, LIHC, LUAD, LUSC, PAAD, TGCT, and THCA. Previous studies showed that the UBE2C expression level involved in cancer progression and invasion.^16^, ^30^, ^39^, ^40^ As well, the result showed that significant high protein expression levels of UBE2C protein in 20 different cancer type increase. However, several samples of gliomas were weakly stained or negative. The variation in UBE2C expression across different tumor types indicates that UBE2C exhibits diverse regulatory mechanisms in distinct types of tumors and is regarded as an oncogene that could be targeted for therapeutic purposes.

Furthermore, the correlation of UBE2C expression with clinical outcomes confirmed that UBE2C overexpression results in poor prognosis, worse overall survival, and disease‐free survival in many cancers. These results were in agreement with previous findings in adrenocortical and oral squamous cell carcinoma.^30^, ^40^ Recent studies showed that the high expression of UBE2C has correlation with poor OS and DFS of cancer patients.^16^, ^30^, ^39^, ^40^ Similarly, according to our study, the high expression of UBE2C was significantly correlated with poor prognosis and shorter OS of patients with ACC, BRCA, KIRC, KIRP, LGG, LUAD, MESO, PAAD, and SKCM. Also, upregulated UBE2C expression leads to worse DFS prognosis of patients with ACC, KIRC, KIRP, LGG, LIHC, MESO, PAAD, PRAD, THCA, UCEC, and UVM.^16^, ^17^, ^41^

Here, we investigated the gene expression profiles of UBE2C in both healthy individuals and cancer patients to evaluate its diagnostic value. Significantly increased UBE2C expression level in various tumor types, including CESC, CHOL, GBM, and UCS tumors (with AUC = 100%) or in ACC, BLCA, BRCA, COAD, ESCA, KICH, KIRC, KIRP, LGG, LIHC, LUAD, LUSC, OV, PAAD, READ, SKCM, STAD, TGCT, and UCEC tumors (with AUC ≥90%) suggest that assessing UBE2C gene expression could serve as a novel biomarker for tumor staging and a promising diagnostic tool prior to symptoms manifestation. The diagnostic value of UBE2C has been previously reported in patients with hepatocellular carcinoma (HCC) and liver cancer.^42^, ^43^ Consistently, a recent study reported that detecting of UBE2C expression level through IHC sections could be an indicator of polyploidy in cancer tissue, allowing discrimination of aggressive subsets of HCC. Thus, UBE2C overexpression is associated with histological diagnosis of polyploidy with the highest sensitivity in HCC and suggesting its potential as a target for the treatment of aggressive HCC subsets.^44^ Furthermore, another study revealed a positive correlation between UBE2C expression levels and tumor stage, local lymph node metastasis, and FIGO stages in endometrial cancer (EC) through IHC examinations. These findings indicated that UBE2C overexpression plays an essential role in diagnosis of EC progression and metastasis, making it a prognostic factor in EC patients.^45^ Overall, our study and previous research highlight the significance of UBE2C as a diagnostic tool for early detection and a potential therapeutic target in various cancers.

Based on our analysis, the highest genetic alteration frequency of UBE2C occurs in colorectal adenocarcinoma and uterine carcinoma, respectively. Likewise, our finding showed that the missense mutation is the major type of UBE2C mutation that happens in COAD, UCEC, bladder carcinoma and sarcoma. In terms of deep deletion, it was dominant alteration among LAML patients. In addition, the OS and DFS were examined and the result showed that UBE2C alteration was related to OS of ESCA and LUSC and DFS of SARC patients. Another study confirmed this in line with us and reported that in 9 cancers including KIRP, ACC, UCEC, MESO, LGG, SARC, COAD, LIHC and HNSC, the mutation in UBE2C is related to poor OS.^41^

Intersectional analysis revealed CDCA3 as an interesting gene, consistently correlated across 33 cancer types. CDCA3 correlation with UBE2C across all TCGA cancers highlights its potential significance in diverse contexts. In a recent investigation led by Song et al. in 2021, associated with ovarian carcinoma a collection of 14 DEGs was pinpointed.^46^ Among these, nine genes, including CDCA3 and UBE2C, were observed to be upregulated and demonstrated significant enrichment in cell cycle regulation as evidenced by gene ontology analysis.^46^ Additionally, when using Kaplan–Meier survival analysis, it became clear that higher levels of CDCA3 and UBE2C together were linked to poorer survival outcomes for patients, regardless of the stage of the tumor or how advanced the tumor appeared under the microscope.^46^ In a separate 2023 study focusing on ACC, 20 oncogenes, such as CDCA3 and UBE2C, were identified by analyzing TCGA and GTEx datasets using ACLBI Web‐based Tools.^30^ This analysis revealed that these 20 oncogenes exhibited elevated expression in tumor tissues and allowed for the investigation of expression differences between tumor and normal tissues.^30^ Notably, this examination singled out UBE2C as a significant DEG between tumor and normal tissues.^30^

We further defined the PPI network of UBE2C; a network consisted 10 nodes, including CCNA2, ANAPC2, CDC16, ANAPC10, UBA1, CDC20, ANAPC11, CDK1, CDC27, and CDC23 and 52 edges. Among the 100 genes showing comparable expression patterns and the 11 interacted genes, we identified three genes, CDC20, CCNA2, and CDK1, that were shared with UBE2C. Interestingly, in an independent study, investigators made a noteworthy discovery using ACLBI Web‐based Tools: UBE2C expression displays a positive correlation with the expressions of CDC20, CDK1, and CCNA2.^30^ This observation signifies the heightened cell cycle advancement characterizing cases of ACC featuring elevated UBE2C expression.^30^ Illustrating another instance of UBE2C and CDC20 co‐expression, Bruno S et al. explored Chronic Lymphocytic Leukemia (CLL), a hematological malignancy where CDC20 has been implicated in the high‐risk category. Their investigation revealed heightened expression of cell cycle‐related genes, notably UBE2C and CDC20.^47^

Remarkably, UBE2C enrichment analyses yielded significant enrichments in 29 gene ontology terms and pathways. The most notable biological process observed was the receptor Anaphase‐Promoting Complex (APC)‐Dependent Catabolic Process. The activation of the APC is facilitated by the regulatory subunit Cdc20 (APC (Cdc20)), which collaborates with UBE2C to direct securin, mitotic cyclins, and other regulatory proteins of the cell cycle for degradation via the proteasome pathway.^48^ According to our findings, the most prominent enriched molecular function is Ubiquitin‐Like Protein Transferase. Furthermore, the Cell Cycle emerged as the most notably significant KEGG pathway. In a research endeavor led by Chen et al., a bioinformatics assessment highlighted the significant involvement of UBE2C in renal cell carcinoma (RCC). They inferred that UBE2C, identified as one of the DEGs, serves as a pivotal element in the cell cycle process, thereby likely regulating the cell cycle pathway in RCC.^49^ In this study, there was a significant correlation between UBE2C expression and most of the immune cells by pan‐cancer analysis. Correspondingly, there was a strong and significant correlation between UBE2C gene expression and all immune cells in THCA. Previous studies have elucidated the dual role of UBE2C in thyroid cancer (THCA) as both a tumor suppressor gene and an oncogene. Inhibition of THCA cell proliferation and induction of apoptosis were observed upon suppression of UBE2C, providing evidence for its oncogenic role. However, the knockdown of UBE2C in THCA cells resulted in increased migration and invasion, upregulation of migration‐related proteins, and increased resistance to sorafenib, suggesting its role as a suppressor gene in THCA.^50^

UBE2C expression has been found to be positively associated with immune checkpoint‐related genes, including CD274, CTLA4, HAVCR2, LAG3, PDCD1, PDCD1LG2, SIGLEC15, and TIGIT in various cancers.^40^, ^51^ Moreover, most of the immune‐related markers and metabolism‐related pathways were associated with UBE2C expression level in THCA.^50^ In hepatocellular carcinoma, UBE2C was found to be positively correlated with the infiltration of regulatory T cells and T follicular helper cells.^52^ In renal cell carcinoma, UBE2C is strongly correlated with the infiltration of various immune cells, including M0 macrophages, regulatory T cells, and CD4+ memory T cells in RCC. These findings suggest that UBE2C may play a role in modulating the immune microenvironment in RCC, and its expression is associated with prognostic significance and tumor progression.^53^ In adrenocortical carcinoma, UBE2C expression is positively linked with T helper Th1 and Th2 cells and negatively correlated with Treg, M2 macrophages.^30^ Although UBE2C overexpression has been linked to immune cell infiltration in various cancer types, it may differ depending on the cancer type and require further investigation.^50^, ^52^, ^54^ Acting as an oncogene in these cancers, UBE2C plays a crucial role in both cancer development and therapy, suggesting its potential as an effective therapeutic target for pan‐cancer. Moreover, studies have highlighted the significance of TMB (tumor mutational burden) as a novel biomarker predicting immunotherapeutic response and its association with prognosis in 13 different cancer types.^51^ Variations in prognosis among cancers may be attributed to tumor heterogeneity. Notably, the expression level of UBE2C is closely linked to TMB. A study has indicated that TMB serves as a valuable biomarker for selecting immune checkpoint blockade (ICB) across various cancer types.^55^ Consequently, the association of UBE2C with immunotherapy suggests its potential as an immunotherapeutic target in the context of these 20 cancer types. Furthermore, UBE2C has been identified as a potential diagnostic and therapeutic biomarker, as well as a prognostic signature for various cancers. The overexpression of UBE2C has been associated with tumor progression^51^, ^56^ and poor prognosis in different cancer types, making it a potential target for immunotherapy. The role of UBE2C in the aggressive progression of various cancers, including tongue squamous cell carcinoma, renal cell carcinoma, thyroid carcinoma, and glioma, has been well‐documented.^50^ These studies provide strong evidence for the significant involvement of UBE2C in the regulation of the immune response and its potential as a therapeutic target in cancer.

Based on the UBE2C ceRNA network, we identified lncRNAs and circRNAs as partners of UBE2C that competitively bind to the associated miRNAs, affecting miRNA‐mRNA interactions. We identified 10 miRNAs as regulatory mediators that could target UBE2C and act as crucial determinants of cancer progression. The functions of these miRNAs depend on specific tumor types. It was reported that miR‐17‐5p promotes proliferation and metastasis of nasopharyngeal carcinoma via targeting p21.^57^ However, other miRNAs, including miR‐615‐3p and miR‐196a‐5p, can act as tumor promotors or suppressors in various cancers.^58^, ^59^, ^60^, ^61^ Interestingly, researchers found miR‐671‐5p and miR‐381‐3p can target FGFR2 to suppress cancer by blocking proliferation and progression in osteosarcoma and esophageal squamous cell carcinoma.^62^, ^63^, ^64^ These data suggest that further in‐depth studies are required to explain the observed heterogeneity according to the type and stage of each cancer.

Given that lncRNAs and circRNAs act as miRNA sponges that hinder binding of miRNAs to their corresponding mRNAs,^65^ we aimed to assess altered transcription patterns in tumors through the evaluation of UBE2C upstream regulators in a ceRNA network. In our study, 21 circRNAs and 128 lncRNAs were identified as selected miRNA regulators, among which the lncRNA nuclear enriched abundant transcript 1 (NEAT1) has been identified as a common regulator of all 10 selected miRNAs. NEAT1‐miRNA regulatory networks play crucial roles in the regulation of tumorigenesis, in which NEAT1 may function either as an oncogene or a tumor suppressor gene.^66^ There is accumulating evidence that NEAT1 facilitates tumor cell growth, migration, and invasion in colorectal cancer via targeting miR‐196a‐5p or miR‐216b or in thyroid carcinoma by regulating miRNA‐214 expression.^67^, ^68^, ^69^ Other researchers have confirmed the oncogenic role of NEAT1 in other tumors, including breast, lung, hepatocellular, ovarian, and prostate cancers.^70^, ^71^, ^72^, ^73^ In addition, higher levels of NEAT1 have been shown to be correlated with poor clinical outcomes^74^ and reduced chemotherapy response in patients.^75^ However, NEAT1 has been demonstrated to act as a protective factor in acute myeloid leukemia (AML).^76^ Therefore, a detailed elucidation of NEAT1‐miRNA regulatory pathways and the impact of their interactions on cancer progression will shed light on the development of novel therapeutic approaches via targeting these potential biomarkers.

## 5 CONCLUSION

In summary, the present study demonstrates that UBE2C is a differential expressed gene in 28 types of cancer, playing a strong prognostic role in OS and DRF of patients with most of the cancers. Also, by evaluating diagnostic value of UBE2C and its correlation with different immune‐related signatures, we conclude that UBE2C is be a theranostic gene, which may serve as a reliable biomarker in diagnosing cancers, improving treatment responses, and increasing the overall survival of cancer patients and can be a promising gene to be target by cancer drugs in the future.

## AUTHOR CONTRIBUTIONS

**Pooya Jalali:** Methodology (lead); writing – original draft (equal);writing – review and editing. **Amir Samii:** Formal analysis (equal); writing – original draft (equal). **Malihe Rezaee:** Writing – original draft (equal). **Arvin Shahmoradi:** Writing – original draft. **Fatemeh Pashizeh:** Writing – original draft. **Zahra Salehi:** Investigation (lead); methodology (lead); supervision (lead); writing – review and editing.

## CONFLICT OF INTEREST STATEMENT

The authors declare no conflict of interest.

## ETHICS STATEMENT

Not applicable.

## Supporting information


**Figure S1.** UBE2C gene expression in different cancer types. With *p* value <.01 and |LogFC| >1, UBE2C was up‐regulated in 30 types of cancer. ACC, Adrenocortical Carcinoma, BLCA: Bladder Urothelial Carcinoma, BRCA: Breast Invasive Carcinoma, CESC: Cervical squamous cell carcinoma and endocervical adenocarcinoma, CHOL: Cholangiocarcinoma, TGCT: Testicular Germ Cell Tumors, COAD: Colon adenocarcinoma, DLBC: Lymphoid Neoplasm Diffuse Large B‐cell Lymphoma, ESCA: Esophageal carcinoma, GMB: Glioblastoma multiforme, HNSC: Head and Neck squamous cell carcinoma, THCA: Thyroid Carcinoma, KICH: Kidney Chromophobe, KIRC: Kidney Renal Clear Cell Carcinoma, KIRP: Kidney Renal Papillary Cell Carcinoma, LAML: Acute Myeloid Leukemia, LGG: Low Grade Glioma, THYM: Thymoma, LIHC: Liver Hepatocellular Carcinoma, LUAD: Lung Adenocarcinoma, LUSC: Lung Squamous Cell Carcinoma, OV: Ovarian Serouscystadeno Carcinoma, PAAD: Pancreatic Adenocarcinoma, UCEC: Uterine Corpus Endometrial Carcinoma, PRAD: Prostate adenocarcinoma, READ: Rectum adenocarcinoma, SARC: Sarcoma, SKCM: Skin Cutaneous Melanoma, STAD: Stomach adenocarcinoma, UCS: Uterine Carcinosarcoma.

**Figure S2.** Diagnostic value of UBE2C in different types of cancer. UBE2C can be a diagnostic factor in CESC, CHOL, GBM, and UCS with AUC = 100%, and ACC, BLCA, BRCA, COAD, ESCA, KICH, KIRC, KIRP, LGG, LIHC, LUAD, LUSC, OV, PAAD, READ, SKCM, STAD, TGCT, and UCEC with AUC ≥90%. ACC, Adrenocortical Carcinoma, BLCA: Bladder Urothelial Carcinoma, BRCA: Breast Invasive Carcinoma, CESC: Cervical squamous cell carcinoma and endocervical adenocarcinoma, CHOL: Cholangiocarcinoma, TGCT: Testicular Germ Cell Tumors, COAD: Colon adenocarcinoma, ESCA: Esophageal carcinoma, GMB: Glioblastoma multiforme, KICH: Kidney Chromophobe, KIRC: Kidney Renal Clear Cell Carcinoma, KIRP: Kidney Renal Papillary Cell Carcinoma, LGG: Low Grade Glioma, LIHC: Liver Hepatocellular Carcinoma, LUAD: Lung Adenocarcinoma, LUSC: Lung Squamous Cell Carcinoma, OV: Ovarian Serouscystadeno Carcinoma, PAAD: Pancreatic Adenocarcinoma, UCEC: Uterine Corpus Endometrial Carcinoma, READ: Rectum adenocarcinoma, SKCM: Skin Cutaneous Melanoma, STAD: Stomach adenocarcinoma, UCS: Uterine Carcinosarcoma.

**Data S1:** Supporting Information.

**Data S2:** Supporting Information.

## Data Availability

The in silico analysis conducted in this study, as well as the datasets presented, are accessible through online repositories. The names of these repositories, along with the accession numbers, are provided in the article.
