# Supplementary figures and images for: UBE2C: A pan‐cancer diagnostic and prognostic biomarker revealed through bioinformatics analysis
Source: Cancer Rep (Hoboken). 2024 Apr 5;7(4):e2032. doi: 10.1002/cnr2.2032 (PMC10995712; doi:10.1002/cnr2.2032)

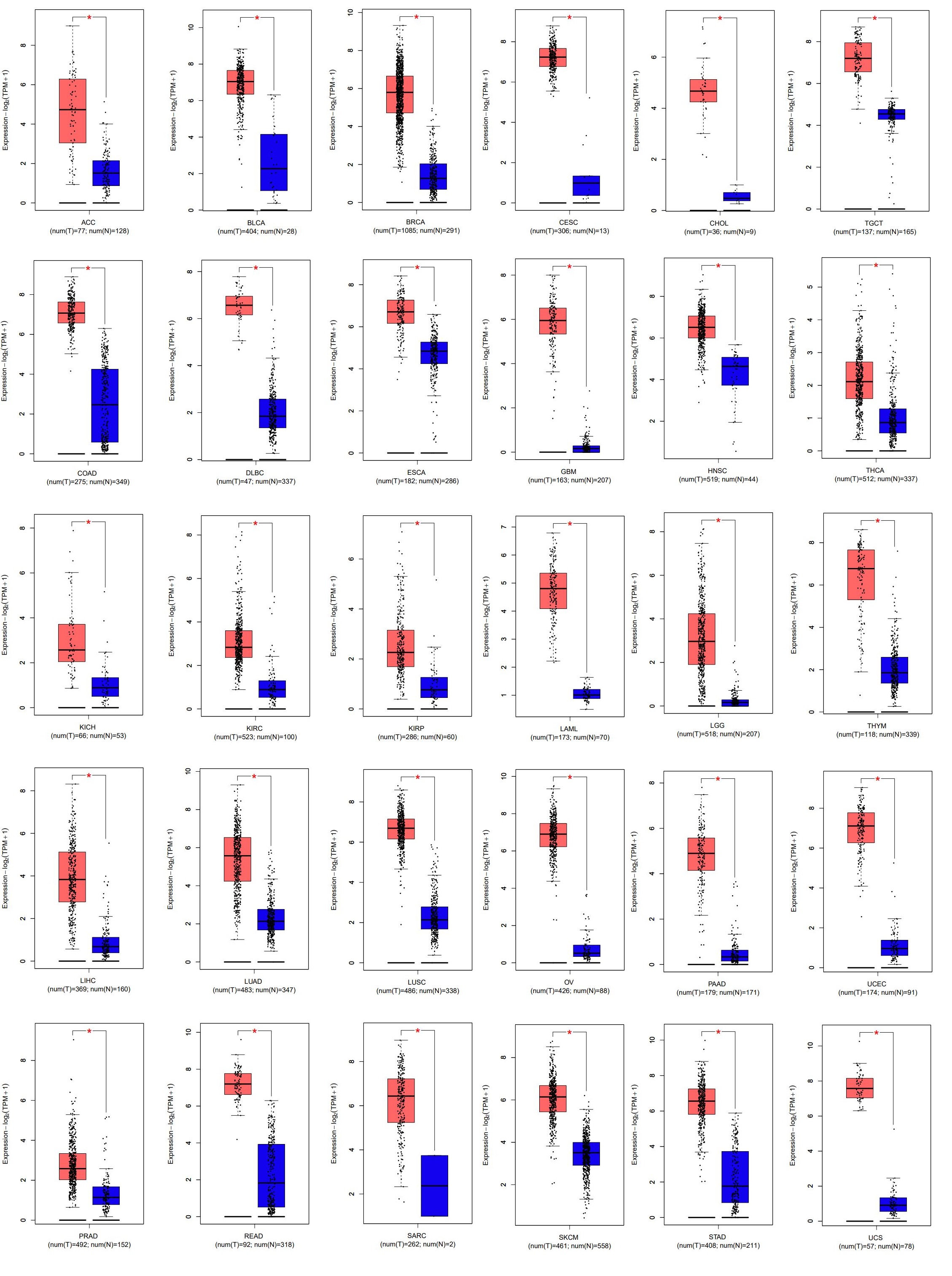

Supplement: Supplementary file 1 — Figure S1. UBE2C gene expression in different cancer types. With p value <.01 and |LogFC| >1, UBE2C was up‐regulated in 30 types of cancer. ACC, Adrenocortical Carcinoma, BLCA: Bladder Urothelial Carcinoma, BRCA: Breast Invasive Carcinoma, CESC: Cervical squamous cell carcinoma and endocervical adenocarcinoma, CHOL: Cholangiocarcinoma, TGCT: Testicular Germ Cell Tumors, COAD: Colon adenocarcinoma, DLBC: Lymphoid Neoplasm Diffuse Large B‐cell Lymphoma, ESCA: Esophageal carcinoma, GMB: Glioblastoma multiforme, HNSC: Head and Neck squamous cell carcinoma, THCA: Thyroid Carcinoma, KICH: Kidney Chromophobe, KIRC: Kidney Renal Clear Cell Carcinoma, KIRP: Kidney Renal Papillary Cell Carcinoma, LAML: Acute Myeloid Leukemia, LGG: Low Grade Glioma, THYM: Thymoma, LIHC: Liver Hepatocellular Carcinoma, LUAD: Lung Adenocarcinoma, LUSC: Lung Squamous Cell Carcinoma, OV: Ovarian Serouscystadeno Carcinoma, PAAD: Pancreatic Adenocarcinoma, UCEC: Uterine Corpus Endometrial Carcinoma, PRAD: Prostate adenocarcinoma, READ: Rectum adenocarcinoma, SARC: Sarcoma, SKCM: Skin Cutaneous Melanoma, STAD: Stomach adenocarcinoma, UCS: Uterine Carcinosarcoma. [file CNR2-7-e2032-s003.JPG]

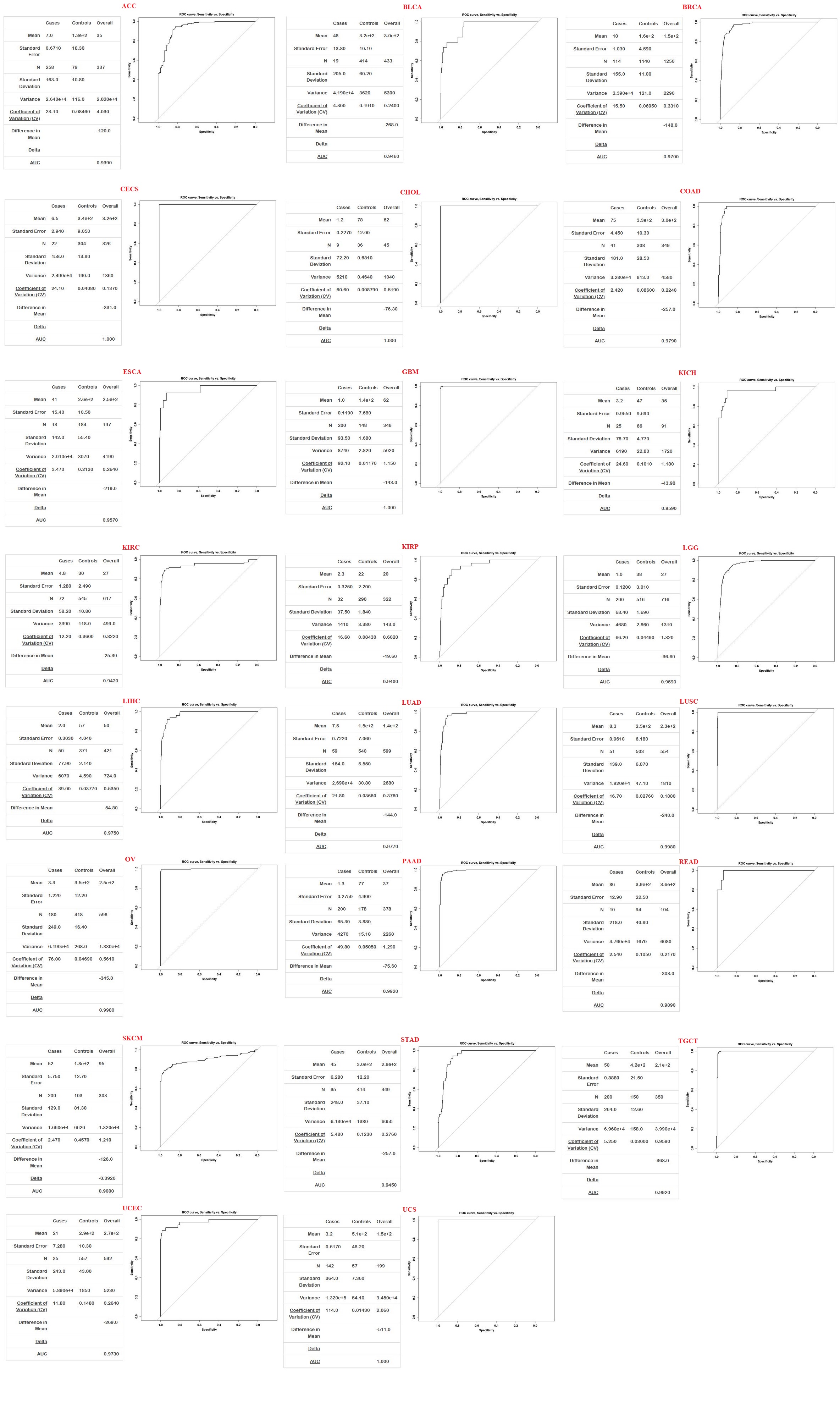

Supplement: Supplementary file 2 — Figure S2. Diagnostic value of UBE2C in different types of cancer. UBE2C can be a diagnostic factor in CESC, CHOL, GBM, and UCS with AUC = 100%, and ACC, BLCA, BRCA, COAD, ESCA, KICH, KIRC, KIRP, LGG, LIHC, LUAD, LUSC, OV, PAAD, READ, SKCM, STAD, TGCT, and UCEC with AUC ≥90%. ACC, Adrenocortical Carcinoma, BLCA: Bladder Urothelial Carcinoma, BRCA: Breast Invasive Carcinoma, CESC: Cervical squamous cell carcinoma and endocervical adenocarcinoma, CHOL: Cholangiocarcinoma, TGCT: Testicular Germ Cell Tumors, COAD: Colon adenocarcinoma, ESCA: Esophageal carcinoma, GMB: Glioblastoma multiforme, KICH: Kidney Chromophobe, KIRC: Kidney Renal Clear Cell Carcinoma, KIRP: Kidney Renal Papillary Cell Carcinoma, LGG: Low Grade Glioma, LIHC: Liver Hepatocellular Carcinoma, LUAD: Lung Adenocarcinoma, LUSC: Lung Squamous Cell Carcinoma, OV: Ovarian Serouscystadeno Carcinoma, PAAD: Pancreatic Adenocarcinoma, UCEC: Uterine Corpus Endometrial Carcinoma, READ: Rectum adenocarcinoma, SKCM: Skin Cutaneous Melanoma, STAD: Stomach adenocarcinoma, UCS: Uterine Carcinosarcoma. [file CNR2-7-e2032-s002.JPG]
